# TRPA1 Antagonists for Pain Relief

**DOI:** 10.3390/ph11040117

**Published:** 2018-11-01

**Authors:** Ari Koivisto, Niina Jalava, Raymond Bratty, Antti Pertovaara

**Affiliations:** 1 Research and Development, Orion Corporation Orion Pharma, POB 425 (Tengströminkatu 8), 20101 Turku, Finland; ari-pekka.koivisto@orionpharma.com (A.K.); niina.jalava@orionpharma.com (N.J.); 2 Research and Development, Orion Corporation, Orion Pharma, POB 6792, Nottingham NG1 1AH, UK; raymond.bratty@orionpharma.com; 3 Department of Physiology, Faculty of Medicine, (Haartmaninkatu 8) POB 63, University of Helsinki, 00014 Helsinki, Finland

**Keywords:** inflammatory pain, neuropathic pain, nociceptive primary afferent nerve fiber, peripheral diabetic neuropathy, spinal cord, transient receptor potential ankyrin 1

## Abstract

Here, we review the literature assessing the role of transient receptor potential ankyrin 1 (TRPA1), a calcium-permeable non-selective cation channel, in various types of pain conditions. In the nervous system, TRPA1 is expressed in a subpopulation of nociceptive primary sensory neurons, astroglia, oligodendrocytes and Schwann cells. In peripheral terminals of nociceptive primary sensory neurons, it is involved in the transduction of potentially harmful stimuli and in their central terminals it is involved in amplification of nociceptive transmission. TRPA1 is a final common pathway for a large number of chemically diverse pronociceptive agonists generated in various pathophysiological pain conditions. Thereby, pain therapy using TRPA1 antagonists can be expected to be a superior approach when compared with many other drugs targeting single nociceptive signaling pathways. In experimental animal studies, pharmacological or genetic blocking of TRPA1 has effectively attenuated mechanical and cold pain hypersensitivity in various experimental models of pathophysiological pain, with only minor side effects, if any. TRPA1 antagonists acting peripherally are likely to be optimal for attenuating primary hyperalgesia (such as inflammation-induced sensitization of peripheral nerve terminals), while centrally acting TRPA1 antagonists are expected to be optimal for attenuating pain conditions in which central amplification of transmission plays a role (such as secondary hyperalgesia and tactile allodynia caused by various types of peripheral injuries). In an experimental model of peripheral diabetic neuropathy, prolonged blocking of TRPA1 has delayed the loss of nociceptive nerve endings and their function, thereby promising to provide a disease-modifying treatment.

## 1. Introduction to TRP Channels

Terminals of nociceptive primary afferent nerve fibers express various types of ion channel receptors that transduce noxious environmental stimuli into electric signals. Thereby, ion channel receptors expressed on nociceptive nerve fiber terminals help to detect harmful stimuli in the environment. Additionally, ion channel receptors expressed on central terminals of nociceptive nerve fibers may amplify transmission of nociceptive signals to central pain-relay neurons. In acute conditions, noxious stimuli detected by ion channel receptors on peripheral terminals induce nociceptive signals that may evoke pain sensations and protective responses, such as axon and flexion reflexes, that help to avoid tissue injury. In pathophysiological conditions, ion channel receptors expressed on nociceptors may contribute to chronic pain and prolonged suffering.

Among ion channel receptors expressed on nociceptors are those belonging to the transient receptor potential (TRP) family that transduce and thereby detect various aspects of potentially harmful stimuli. The best-known TRP channel is transient receptor potential vanilloid 1 (TRPV1) that has unique sensitivity to capsaicin but it is also activated by noxious heat. Since there are several recent reviews on the role of TRP channels in general or TRPV1 channels in particular in pain treatment (e.g., [1,2,3,4]), this brief review focuses only on one member of the TRP family that according to a number of studies is implicated in pain, transient receptor potential ankyrin 1 (TRPA1) as a target for pain relief.

## 2. TRPA1 in Transduction of Noxious Stimulation

TRPA1 is a calcium-permeable nonselective cation channel [5,6]. Biophysical and structural properties of the TRPA1 channel are outside the scope of this article and have been thoroughly reviewed elsewhere (e.g., [7,8,9,10,11]). In the nervous system, TRPA1 is expressed on a subpopulation of TRPV1-expressing nociceptive primary afferent neurons [5], astrocytes [12], oligodendrocytes [13], and on peripheral analogs of oligodendrocytes, Schwann cells [14]. In addition, among non-neuronal cells expressing TRPA1 are epidermal keratinocytes, endothelium, and vascular smooth muscle [15].

Originally, TRPA1 was considered to detect noxious cold and thereby cold pain [5]. However, later studies suggested that TRPA1 is involved in detecting cold hypersensitivity rather than physiological cold pain [16]. Instead, there is abundant evidence indicating that TRPA1 is activated by various types of irritant chemicals that include endogenous compounds produced by tissue injury. Among the numerous chemicals activating TRPA1 are some spices (e.g., cinnamon, mustard oil, and wasabi), formalin, reactive oxygen species (ROS), reactive nitrogen species, reactive carbonyl species, cannabinoids, volatile anesthetics, etc. [7]. Thereby, TRPA1 channels expressed on nociceptors are considered to be important as chemosensors of nociception. 

Previously, mammalian TRPA1 was not considered to be a detector of noxious heat, unlike TRPA1 in *Drosophila* or rattle snakes [17]. However, recently it was reported that after redox modification and exposure to some ligands human TRPA1 could be activated by heat [18]. Moreover, another recent study showed that the detection of acute noxious heat stimuli in mice depends on three functionally redundant TRP channels that include TRPA1 as well as TRPM3 and TRPV1 [19]. This finding may explain why pharmacological or genetic blocking of the TRPA1 channel alone has not induced changes in heat nociception in most of the earlier mammalian studies. 

Responses of nociceptive primary afferent nerve fibers to noxious mechanical stimulation have been attenuated by pharmacological or genetic blocking of the TRPA1 indicating that peripheral TRPA1 channels contribute to mechanical nociception [20,21]. Sensory neuron-specific deletion of TRPA1 produced attenuation of the limb withdrawal response evoked by mechanical stimulation [22]. While this finding is in line with the hypothesis that TRPA1 expressed on sensory nerve fibers exerts a role in transduction of mechanical pain, it leaves open the possibility that the sensory neuronal TRPA1 interacts with other transducer molecules on the neuronal membrane to evoke a mechanically-induced sensory signal. Moreover, it should be noted that keratinocytes that are known to have a role in nociception [23] and that also express TRPA1 [21,24] may also contribute to mechanical nociception.

Central mechanisms have been traditionally considered to be important in mechanical hypersensitivity, unlike in heat hypersensitivity [25]. Thereby, when considering the potential contribution of TRPA1 to mechanical hypersensitivity that may to a large extent be dependent on central mechanisms, one needs to keep in mind the TRPA1-mediated amplification of transmission in the spinal dorsal horn [26]. However, there is accumulating evidence indicating that also peripheral mechanisms, including those involving TRPA1, may also contribute to mechanical hypersensitivity. In line with this, mechanically evoked responses were facilitated by inflammation only in a population of primary afferent nerve fibers expressing TRPA1 [27], and mechanical sensitization of nociceptors was attenuated by pharmacological blocking of TRPA1 [28]. Immune cell-to-sensory neuron signaling was recently shown to be among peripheral TRPA1-mediated mechanisms of nociceptor sensitization [29]. This included activation of the type 2 angiotensin II receptor on peripheral macrophages which triggers production of reactive oxygen/nitrogen species leading to TRPA1-mediated nociceptor sensitization. One more peripheral TRPA1-mediated mechanism contributing to mechanical hypersensitivity following nerve injury involves Schwann cell TRPA1. This has been proposed to orchestrate neuroinflammation and oxidative stress that promote nociception [14].

## 3. TRPA1 in Secondary (Central) Hyperalgesia

After skin injury, the intact area surrounding the injury may be sensitized to innocuous as well as noxious mechanical stimuli, leading to tactile allodynia and mechanical hyperalgesia, respectively [25]. The sensitization of intact skin surrounding the injury area has been called secondary hyperalgesia, and it is typically observed with mechanical rather than thermal stimuli and central mechanisms are involved [25]. In experimental animals, mechanical hyperalgesia in the intact skin area adjacent to injury was reversed following intrathecal administration of a TRPA1 antagonist indicating that spinal TRPA1 expressed on central terminals of primary afferent nerve fibers contributes to secondary hyperalgesia [30,31]. Moreover, cutaneous blood flow response adjacent to the skin injury was reduced following intrathecal administration of TRPA1, suggesting that spinal TRPA1 is involved in the dorsal root reflex that through antidromic activation of nociceptive primary afferent nerve fibers contributes to cutaneous neurogenic inflammation [32]. In line with these experimental animal results, a study in humans showed that a gain-of-function mutation in TRPA1 increases secondary hyperalgesia and neurogenic inflammation adjacent to an injury site [33].

A plausible mechanism explaining the contribution of spinal TRPA1 to central hyperalgesia is that injury activity induces ROS in the spinal cord dorsal horn [34]. At least partly, ROS is released from spinal cord microglia [35]. ROS is an established endogenous agonist of TRPA1 [36] that, on central endings of nociceptive primary afferent nerve fibers, amplifies glutamate-mediated transmission to spinal pain-relay neurons [26]. Additionally, injuries have been shown to induce spinal generation of various other endogenous TRPA1 agonists, such as hepoxilin A3 or 5,6-EET [37]. However, among cells expressing TRPA1 is also the astrocyte [12]. Injury-induced activation of spinal astrocytes has been shown to contribute to pain hypersensitivity due to coupling to adjacent astrocytes or neurons, which may promote the spread of excitation and thereby facilitate pain [38]. In line with this possibility, gliogenic long-term potentiation in the spinal cord has been reported to exert a role in wide-spread pain hypersensitivity following injury discharge [39].

## 4. TRPA1 in Sleep Deprivation-Induced Pain Hypersensitivity

Loss of sleep may intensify pain in the clinic and cause pain hypersensitivity in experimental conditions [40]. Vice versa, pain may cause sleep disturbances. Spinal administration of TRPA1 antagonists in the rat has reversed pain hypersensitivity induced by sleep deprivation indicating that spinal TRPA1 contributes to sleep deprivation-induced pain hypersensitivity [31]. This result supports the hypothesis that sleep deprivation induces spinal generation of ROS through descending pathways. This is potentially due to an effect on spinal astrocytes. In agreement with this, some of the descending pathways have been shown to activate an NMDA-nitric oxide cascade [41] that has been associated with activation of astrocytes in the spinal cord dorsal horn [42]. In activated spinal astrocytes, D-amino acid oxidase (DAAO) generates hydrogen peroxide, which, through its effect on TRPA1 on the central endings of nociceptive primary afferent nerve fibers, may amplify transmission of pain-related signals from primary afferent nerve fibers to central neurons of the pain pathways. This proposal is in line with the observations that the enhancement of pain-related behavior following sleep loss was attenuated not only by a spinally administered TRPA1 antagonist but also by spinal administration of carbenoxolone, an inhibitor of astrocytes [43], and by spinal administration of DAAO inhibitors [44]. Collectively, these results indicate that spinal TRPA1 is a final common pathway for a pain-promoting cascade induced by sleep loss. This proposal, however, does not exclude a parallel contribution of other mechanisms promoting pain behavior. Based on experimental animal studies, it may be hypothesized that TRPA1 antagonists could have a dual beneficial effect in some clinical pain conditions that are associated with sleep disturbances. First, blocking TRPA1 may attenuate pain that promotes sleep loss. Second, TRPA1 block is expected to inhibit the pronociceptive circuitry driven by sleep loss.

## 5. Peripheral (Painful) Diabetic Neuropathy

Peripheral (painful) diabetic neuropathy (PDN) is a common consequence of diabetes mellitus. PDN may produce symptoms that vary from paraesthesiae and sustained pain to sensory deficits. Small fiber functions (such as detection of pain and warmth) are affected early, and large fiber-mediated functions (such as detection of vibrotactile stimuli) are affected later [45]. PDN is known to have multiple mechanisms. There is accumulating evidence indicating that one of the earliest mechanisms contributing to PDN is that involving TRPA1 [46]. In line with this, diabetes mellitus produces endogenous substances, such as methylglyoxal and 4-hydroxy-nonenal [47,48] that are TRPA1 agonists [36].

Endoplasmic reticulum (ER) stress is present in diabetic peripheral neuropathy [49]. Experimental induction of ER stress with tunicamycin was shown to drive neuropathic pain-like behavior without inducing hyperglycemia [50]. Interestingly, pharmacological block of TRPA1 was able to reverse tunicamycin-induced mechanical hypersensitivity [51]. ER stress is, therefore, a plausible candidate mechanism for initiation and perhaps also for maintenance of neuropathic pain in early phase of painful diabetic neuropathy. In the long term, release of diabetes-generated compounds may cause a sustained influx of calcium ions through TRPA1 channels in nerve terminals [48]. This is expected to result in axoplasmic calcium dysregulation and excitotoxicity [52]. Thereby, the sustained TRPA1 channel-mediated influx of calcium provides a possible early mechanism for dysfunction and loss of small fiber endings. In agreement with this hypothesis, treatment with a TRPA1 antagonist delayed the loss of substance P-like immunoreactivity-expressing small fiber endings and their nociceptive function in diabetic animals [48]. In another experimental animal study, an increased concentration of methylglyoxal caused by a glyoxylase inhibitor led to a pain phenotype in wild-type but not TRPA1 knockout animals. This finding is in line with the proposal that a substance produced in diabetes, methylglyoxal, contributes to the TRPA1-mediated development of PDN symptoms [53]. These experimental findings are in agreement with clinical observations demonstrating that methylglyoxal is increased both in type 1 and type 2 diabetes [54] and that an increased level of methylglyoxal is associated with the development of PDN symptoms [55,56]. In addition to TRPA1, Na_v_1.8 is among ion channels that may contribute to the methylglyoxal-induced pain behavior in diabetic animals [55,57].

While TRPA1 expressed on distal nociceptive nerve endings exerts an important role in the diabetes-induced structural and functional changes of small diameter nerve fibers, amplification of nociceptive signals by spinal TRPA1 is likely to contribute to the induction and preservation of pain facilitation in diabetic animals. This is suggested by the finding that while a systemically administered TRPA1 antagonist effectively attenuated the maintenance of pain facilitation in diabetic animals [58], a considerably lower dose of a TRPA1 antagonist was needed to attenuate diabetic pain hypersensitivity following intrathecal than systemic or intraplantar application [30]. In line with this, intraplantar injection of methylglyoxal recapitulated pain behavior observed in diabetic neuropathy and was associated with activation of nociceptive spinal dorsal horn neurons [59].

**Figure 1:**
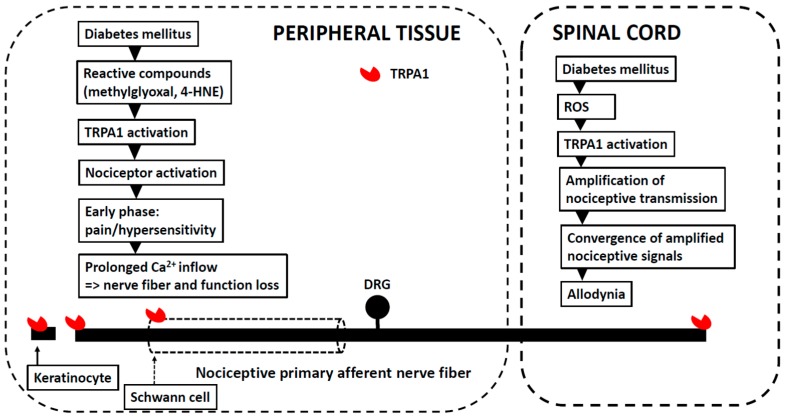
A schematic figure showing potential sites of action for TRPA1 in peripheral diabetic neuropathy (PDN). In the **left** (peripheral tissue), a possible cascade generated in diabetes that drives TRPA1 on nociceptive nerve endings, leading first to excitation that is followed by a loss of TRPA1-expressing nociceptive nerve fiber endings and their function. Note also that activation of TRPA1 on keratinocytes and/or Schwann cells might contribute to facilitation of pain behavior. In the **right** (spinal cord), a possible cascade that drives TRPA1 on central terminals of nociceptive primary afferent nerve fibers in diabetic animals, leading to enhanced transmission that, through central convergence of nociceptive signals to e.g., wide-dynamic range neurons, might contribute to allodynia. DRG: dorsal root ganglion.

Together, the above findings imply that while a TRPA1 antagonist, the effect of which is restricted to the periphery, delays the development of experimental diabetes-induced early anatomical and functional changes in TRPA1-expressing peripheral nociceptors, a peripherally restricted block of TRPA1 is expected to have a considerably weaker effect against diabetes-induced pain behavior than a TRPA1 antagonist that spreads also to the central nervous system (particularly to the spinal cord) to attenuate TRPA1-mediated amplification of nociceptive transmission in diabetes (Figure 1).

A recent mouse study showed that TRPA1 is involved in cold hypersensitivity while the diabetes-induced late mechanical hyposensitivity or the loss of PGP9.5-sensitive intraepidermal nerve fibers were not dependent on TRPA1, as revealed by a comparison of diabetic wild-type and TRPA1 knockout animals [60]. Pan-neuronal marker PGP9.5 labels various types of sensory nerve fibers, including those that do not express TRPA1 and that are involved in signaling mechanically evoked messages. Therefore, this finding supports the proposal that TRPA1-independent metabolic mechanisms are involved in causing mechanical hyposensitivity and destruction of mechanoreceptive nerve fibers in prolonged diabetes. In the early phase of diabetes, in contrast, endogenous TRPA1 agonists may have a key role in the induction of the first symptoms and degenerative signs of PDN through action on a subpopulation of TRPA1-expressing nociceptive nerve fibers. Consequently, blocking the TRPA1 channel promises to be a mechanism-based treatment that can delay the development of PDN. With prolongation of diabetes, metabolic mechanisms are expected to exert a major role in PDN leading to a loss of various types of nerve fibers and mechanical hyposensitivity that are independent of TRPA1, as demonstrated in a recent mouse study [60].

### Other Aspects Related to Experimental Models of PDN

The streptozotocin (STZ)-induced rodent model has been commonly used in diabetes studies. It should be noted that STZ itself has been shown to activate TRPA1, which should be taken into consideration in the interpretations particularly when dealing with acute effects of STZ [61]. However, since single acute injection of STZ is enough to produce diabetes that lasts for months and STZ is eliminated from the circulation within a couple of hours [62], it does not seem likely that TRPA1-mediated effects that take place several weeks after the induction of diabetes could be explained by direct actions of STZ on TRPA1. 

Local injection of STZ to brain ventricles is used to model cognitive dysfunction, which is increasingly recognized as type 1 and 2 diabetes-related symptom [63]. Interestingly, STZ injection was shown to cause selective injury to myelin and axons but not systemic hyperglycemia [64]. It is possible that local activation of TRPA1 by STZ in myelinating oligodendrocytes drives white matter injury in such a model. In agreement with this, genetic deletion of *TRPA1* from birth was shown to reduce myelin basic protein and alter myelination profile in mouse brain [65]. In the central nervous system, increased methylglyoxal following induction of experimental diabetes has been suggested to be a cause for neurotoxic effects such as increased apoptosis of hippocampal neurons that has been associated with cognitive dysfunction [66]. TRPA1 expressed on oligodendrocytes might play a role also in the methylglyoxal-driven hippocampal damage observed in animals with experimental diabetes.

Muscle cramps are a common symptom in diabetes [67,68] and motoneuron injury is present in an STZ-induced animal model of diabetes [69]. Recent human TRPA1 gain-of-function mutation carriers suffered from a number of typical TRPA1 overactivation-related symptoms but cramp-fasciculation syndrome was a novel symptom not previously associated with TRPA1 [70]. Interestingly, TRPA1 is expressed also in human motoneurons [24]. This clinical genetic finding raises the question whether a centrally acting TRPA1 antagonist might have potential to treat cramp-fasciculation symptoms.

## 6. Peripheral Traumatic Neuropathy

Peripheral nerve injuries are known to induce pain and hypersensitivity [45]. Pain hypersensitivity induced by peripheral nerve injury has been attenuated by blocking TRPA1 [71,72]. Amplification of the pain-related signal transmission by spinal TRPA1 contributes to neuropathic hypersensitivity. This is indicated by the observation that a low intrathecal dose of a TRPA1 antagonist effectively reduced hypersensitivity in animals with a spinal nerve ligation-induced neuropathy [31]. In animals with spinal nerve ligation-induced neuropathy, TRPA1 mRNA was down-regulated in the ligated nerve and up-regulated in the adjacent intact nerves [73], although TRPA1 mRNA results following peripheral nerve injuries have been variable (see for references in [74]). Interestingly, it was earlier reported that intact nerves adjacent to the injured nerve may contribute to neuropathic pain [75] providing a potential explanation for the up-regulation of TRPA1 mRNA in intact nerves.

The amygdala, a key structure for primary emotions, is also involved in processing and control of pain. Recently, it was reported that amygdaloid microinjections of antioxidants or selective TRPA1 antagonists attenuated mechanical hypersensitivity and pain affect in nerve-injured animals [76]. While immunocytochemical and molecular biological evidence for amygdaloid TRPA1 expression in neuropathic animals is still missing, this pharmacological finding suggests that nerve injury induces oxidative stress and potentially amygdaloid expression of TRPA1 in neuropathy. Furthermore, the finding suggests that a systemically administered TRPA1 antagonist might attenuate processing of nociception and pain affect in peripheral neuropathy at multiple levels of the neural system.

## 7. Chemotherapy-Induced Neuropathy

A common side-effect of drugs used for the chemotherapy of cancer is neuropathy that is associated with severe cold and mechanical allodynia. In experimental animal studies, blocking TRPA1 has effectively attenuated cold and mechanical allodynia induced by anti-cancer drugs, such as paclitaxel or oxaliplatin, indicating that TRPA1 is involved in chemotherapy-induced neuropathy [77,78]. Concerning potential clinical applications, a promising experimental animal finding is that a brief prophylactic treatment with a TRPA1 antagonist before start of the chemotherapy effectively prevented the development of the chemotherapy-induced neuropathy [79].

## 8. Osteoarthritis

Osteoarthritis is a common disorder that causes pain in joints. Osteoarthritis typically causes destruction of articular cartilage, formation of new bone in the subchondral region, and formation of new bone and cartilage at the joint margins. In experimental animal studies, monosodium iodoacetate is commonly used to induce osteoarthritis. In animals with experimental osteoarthritis, blocking pharmacologically TRPA1 attenuated mechanical hypersensitivity in nociceptive neurons of the spinal dorsal horn [80], while TRPA1 block did not attenuate sustained pain as revealed by a conditioned place preference test [81]. These results indicate that TRPA1 has a more important role in mechanical hypersensitivity than ongoing pain in osteoarthritic animals. Pharmacological or genetic TRPA1 block has attenuated not only hypersensitivity but also inflammation induced by osteoarthritis [82], although not in all experimental conditions [83]. Recently, TRPA1 was shown to be functionally expressed in primary human osteoarthritic chondrocytes that possibly explains some of the symptoms of osteoarthritis and provides a potential drug treatment target [84]. 

## 9. Postoperative Pain

Surgical operations induce ongoing pain and hypersensitivity of the operated area. Pharmacological block of TRPA1 has attenuated guarding behavior, an index of sustained pain, and mechanical hyperalgesia in a rat model of postoperative pain induced by skin and muscle tissue incision in the paw [85]. Local administration of TRPA1 antagonists into the operated area attenuated guarding behavior and mechanical hyperalgesia, while administration of TRPA1 antagonists into the contralateral control area had no effect on pain behavior. This finding indicates that the critical site for the pain suppression induced by pharmacological block of TRPA1 is the peripheral injury site. Moreover, spinal TRPA1 was involved in mechanical allodynia (i.e., hypersensitivity to low intensity stimuli), since it was attenuated by spinal administration of TRPA1 antagonists [85]. This result resembles that obtained in neuropathy models in which spinal TRPA1 was involved in mediating mechanical allodynia-like symptoms [30,31]. It may be speculated that, in addition to peripherally mediated hyperalgesia and ongoing pain, postoperative pain model can induce a central neuropathic pain-like component that is expressed as spinal TRPA1-mediated mechanical allodynia. A mouse study in which only skin was incised, however, reported that TRPA1 knockout does not attenuate mechanical hypersensitivity [86]. Among possible explanations for this difference in results is a species difference (rat versus mouse), a difference in the incised tissue (skin + deep tissue incision versus skin incision), and other differences in the experimental procedures (e.g., pharmacological block versus knockout of TRPA1). A recent rat study showed that both superficial and deep skin incisions increase release of endogenous TRPA1 agonists (ROS and H_2_O_2_), but TRPA1 exerts a significant role in postoperative pain behavior only following deep skin incision that is most prominently seen as attenuation of guarding of the injured paw following topical administration of a TRPA1 antagonist [87]. 

## 10. Pain and Inflammation Induced by Bacterial Infection

The complete form of Freund´s adjuvant (CFA), a mixture that contains inactivated and dried mycobacteria, causes acute pain, mechanical allodynia, swelling of tissue and chronic inflammation when injected to the paw or knee. Several studies have demonstrated that the CFA-induced inflammatory pain can be reduced by administration of selective TRPA1 antagonists or knockout of TRPA1 [72,80,83,88,89,90]. This result indicates that bacteria can directly or indirectly drive TRPA1-expressing sensory neurons and thereby cause pain and inflammation.

The question how microbes activate sensory neurons expressing TRPA1 has been addressed in two elegant studies [91,92]. In one of them, it was shown that live bacterial load rather than tissue swelling or immune activation correlates with hyperalgesia or pain in a mouse model of infection induced by Staphylococcus aureus, a frequent cause of wound infections in humans [91]. The study showed that bacteria can drive sensory neurons by a direct action. Moreover, activation of sensory neurons was mediated by N-formyl peptides and alpha-hemolysin, substances that are released from bacteria. The other investigation addressing the interaction of TRPA1 and microbes showed that lipopolysaccharide (LPS) that is a typical cell wall constituent of Gram negative bacteria, can directly drive TRPA1 and thereby induce pain and inflammation [92]. This observation is interesting since it has been reported that toll-like receptor 4 (TLR-4) of the innate immunity system, that was earlier regarded as the major LPS receptor, was not necessary for such an effect. Moreover, the TRPA1-related neurogenic inflammation was enhanced by concurrent administration of LPS with another agonist of TRPA1 such as 4-hydroxynonenal. This type of an additive effect may be highly pertinent in vivo, when multifarious inflammatory TRPA1 agonists are present and their levels are increased. However, the LPS-induced hypothermia is not mediated by TRPA1, since knockout of TRPA1 did not influence it [92].

Low grade metabolic endotoxemia (i.e., an increased blood plasma level of LPS) is prevalent in metabolic syndrome and diabetes [93]. Accumulating evidence indicates that following an excessive high-fat diet, gut microflora may act as a source of an increased systemic level of LPS [94]. An increase in the systemic LPS level may enhance pain in diabetes and advance development of neuropathy, since LPS is a TRPA1 agonist and it enhances the effect of other TRPA1 agonists such as 4-hydroxynonenal, the concentration of which is increased in diabetes.

Low back pain is one of the most common chronic pain conditions and is often difficult to treat. Pathophysiological mechanisms of low back pain are not well understood. One proposed back pain mechanism is a chronic low grade bacterially induced inflammation. In a double-blind randomized clinical trial, low back pain treatment for 100 days with antibiotics improved significantly the primary outcomes, disease-specific disability and low back pain as well as a number of secondary outcomes [95]. This result supports the hypothesis that chronic low grade bacterial inflammation contributes to pathophysiology of low back pain. Moreover, patients with low back pain caused by lumbar disc herniation were recently shown to have elevated plasma levels of methylglyoxal that is an endogenous TRPA1 agonist [96]. Taking into account the potential role of bacterial interaction with TRPA1 in generation of pain [91,92], an intriguing question is whether patients with low back pain might benefit from a treatment with a selective TRPA1 antagonist that does not have the risks of a long-term antibiotic treatment.

## 11. Migraine

Migraine patients have episodic headache attacks that are typically unilateral. The pain reaches its maximum within a few hours and is associated with nausea and vomiting. Headache attacks in migraine patients typically last from one or two hours up to three days. Migraine can be accompanied by increased sensitivity to light, sound, movement, smell and it frequently causes nausea and vomiting. About one third of migraine patients experience short-lived aura, such as colored flashes and transient visual field defects, before onset of migraine attack.

So far, no clear endogenous cause of migraine headache has been established. However, a number of exogenous triggers have been described that include cigarette smoke, exposure to glyceryl trinitrate, a variety of odors and excessive use of some drugs. The wide variety of chemicals that are known to trigger migraine present an arduous challenge to understand the molecular mechanisms inducing migraine attack.

Changes in the cranial blood flow are tightly associated with migraine attacks implying that neurovascular mechanisms play an important role in migraine. Nociceptive substance P- and calcium gene-related peptide (CGRP)-expressing unmyelinated nerve fibers originating from trigeminal ganglion innervate large cerebral vessels, pial vessels, large venous sinuses and dura mater in the cranium. Activation of these afferent nerve fibers conveys nociceptive signals to the brain but the release of CGRP induces also neurogenic inflammation in the peripheral tissue [97]. Capsaicin-sensitive (i.e., TRPV1-expressing) nerve fibers have been shown to elicit meningeal vasodilation suggesting that they may exert an important role in meningeal nociception [98]. Taking into account that TRPA1 is co-localized in a subpopulation of TRPV1-expressing neurons, this result supports the hypothesis that meningeal TRPA1 may also contribute to migraine pain [99]. Additionally, the finding that a wide diversity of chemical compounds are TRPA1 agonists is in line with the proposal that TRPA1 in trigeminal neurons may be a common mechanism for a wide variety of migraine triggers. In line with this, a number of migraine triggers have been identified as TRPA1 activators and a number of drugs used for migraine treatment desensitize or inhibit TRPA1 [100]. Furthermore, TRPA1 activation causes release of CGRP that is considered a pro-migraine peptide [100].

Migraine attacks are more common in patients who have experienced infantile colic in their early life than in those who do not have a history of infantile colic. This finding supports the hypothesis that colic may be a form of migraine attack or that colic may prime for future migraine attacks [101]. Interestingly, TRPA1 is the last among the TRP channels in sensory neurons of rodents that is expressed during development [102]. Based on this finding, it may be speculated that human TRPA1 expression coincides temporally with the emergence of colic symptoms providing a possible link between TRPA1 activation and induction of colic symptoms. TRPA1 expression must be seen as a substrate and a presumably food-borne or endogenous TRPA1 agonist as a trigger for colic, since not all children suffer from colic.

Intranasal exposure to environmental and cigarette smoke irritant acrolein and some other TRPA1 agonists have been shown to induce neurogenic inflammation, increase meningeal blood flow and CGRP release [103]. Moreover, these actions were prevented by selective antagonists of CGRP or TRPA1. These findings indicate that exposure to various TRPA1 agonists in the environment can induce migraine-like symptoms. Umbellulone is another irritant in the environment and an established migraine trigger that has also been shown to drive human and rodent TRPA1 channels in vitro, evoke neurogenic vasodilation and inflammation as well as migraine-associated hypersensitivity [104]. These effects were prevented by HC-030031, a selective TRPA1 antagonist, and by sumatriptan, a commonly used migraine drug [104].

Cortical spreading depression is a cellular depolarization wave that travels across the cerebral cortex and that has a correlation with migraine aura [105]. It has been shown that cortical spreading depression generates oxidative stress and thereby causes activation of TRPA1 in trigeminal ganglia [106]. Local application of H_2_O_2_, an established TRPA1 agonist, to the meninges induced electrical spiking activity in sensory neurons which is a sign of pronociception. The H_2_O_2_-induced increase of excitability of trigeminal sensory neurons was mediated by action on TRPA1 as indicated by reversal of the effect by a selective TRPA1 antagonist TCS-5861528 [106]. In agreement with this, a recent study using brain slices demonstrated that the spread of cortical depression was facilitated by a TRPA1 agonist and delayed by a block of TRPA1 [107].

In addition to compounds generated in oxidative stress, it may be predicted that there are multiple mechanisms of migraine-like headache that involve TRPA1 activation in meningeal sensory neurons. Among the candidates is nitroglycerin-induced migraine in which TRPA1 is directly driven by nitric oxide [108]. A recent experimental animal study showed that while nitric oxide is involved in initiation of periorbital allodynia and vasodilatation induced by exposure to glyceryl nitrate, a well-known migraine trigger, only vasodilatation was shown to be maintained by a direct nitric oxide action, whereas maintenance of allodynia proved to be a neuronal event mediated by TRPA1 activation and ensuing oxidative stress [109]. Another member of gasotransmitter family that presumably has a role in migraine, hydrogen sulfide, was shown to activate nociceptive trigeminal neurons partly due to action on TRPA1 [110] potentially providing one more migraine mechanism involving TRPA1.

At least partly, headache in bacterial meningitis might depend on leakage of LPS and formyl peptides from bacteria and cells since these substances have been shown to excite sensory neurons expressing TRPA1 [91,92]. Mitochondrial encephalopathy that is associated with lactic acidosis and stroke-like episodes (MELAS) might also involve TRPA1-mediated headache mechanisms [111], since lactic acidosis-induced acidosis should activate human TRPA1 [112].

There is accumulating evidence indicating that genetic and epigenetic factors play a role in explaining individual differences in migraine vulnerability and underlying pathophysiological mechanisms. For example, TRPM8 was strongly linked to migraine in a human genome-wide association study [113], while genome-wide studies have not revealed such a link between TRPA1 and migraine. However, differential TRPA1 expression has been reported to be linked to individual heat pain sensitivity in discordant twins and healthy volunteers. This was indicated by the finding that increased methylation of the TRPA1 gene promoter correlated with the increased expression of TRPA1 in tissues and the increased heat pain sensitivity [114]. This observation indicates that epigenetic changes causing increased TRPA1 expression in sensory neurons may amplify heat nociception in affected individuals, although under physiological conditions TRPA1 is not directly activated by heat. In line with this, heat pain sensitivity is increased between migraine attacks in episodic and chronic migraine patients [115]. It still remains to be studied whether migraine patients and/or infants suffering from colic pain have a hypermethylated TRPA1 promoter and increased TRPA1 expression.

There is still a huge need for more effective treatment of migraine. Compelling evidence supports the proposal that TRPA1 antagonists may provide a promising future alternative for the prevention and acute treatment of migraine. 

## 12. Visceral Pain

Pain of visceral origin, such as irritable bowel syndrome or chronic pancreatic pain, is still a challenge to treat in the clinic. There is accumulating evidence from experimental animal studies indicating that TRPA1 is expressed in the viscera and that it contributes to visceral pain (see for reviews [116,117]). For example, experimental inflammatory hyperalgesia of the colon was associated with up-regulation of TRPA1 and hyperalgesia was reduced by spinal administration of TRPA1 antisense oligodeoxynucleotide [118,119]. Experimental results on pancreatic pain indicate that TRPA1 mediates chronic pancreatic inflammation and pain. This is shown by the finding that a chemical that induces strong pancreatitis when administered into the pancreatic duct of wild-type mice had a markedly weaker effect in the pancreatic duct of TRPA1 knockout animals [120]. Importantly, early administration of a TRPA1 or a TRPV1 antagonist attenuated the transition and development of experimental pancreatic inflammation and pain from acute experimental to chronic, thereby providing an effective disease-modifying therapy [121].

## 13. TRPA1 Agonists for Pain Treatment

There is preclinical evidence suggesting that TRPA1 agonism may contribute to the analgesic effect induced by acetaminophen (paracetamol). This was shown by the finding that the reactive metabolite of acetaminophen acts in vivo as a TRPA1 agonist and thereby attenuates pain behavior through desensitization of sensory neurons expressing TRPA1 [122]. This result raises an intriguing question whether TRPA1 agonism may be a general approach for developing new analgesic compounds.

It is easier to identify a novel TRPA1 agonist than a novel TRPA1 antagonist scaffold. While this could be seen as an advantage for a drug development approach, several major challenges can be foreseen from the drug discovery point of view when developing drugs, the effect of which is based on TRPA1 agonism. First, the TRPA1 agonist-induced long-term sustained activation and possible pore dilation of TRPA1 channel is expected to result in excessive inflow of calcium and consequently, excitotoxicity [48]. Second, most of the identified TRPA1 agonists are reactive substances. This raises questions about risks associated with reactive compounds and their metabolites that may lead to idiosyncratic adverse drug reactions. Third, in several pathophysiological pain conditions, generation of several chemically diverse endogenous TRPA1 agonists is increased. Before a synthetic agonist of TRPA1 is used for pain treatment, the possibility that it enhances rather than reduces pain needs to be excluded. Therefore, it might be argued that developing an antagonist or negative allosteric modulator of TRPA1 could be a more straightforward and successful way to develop new drugs for the treatment of pain conditions that involve contribution of TRPA1.

## 14. TRPA1 Antagonists

Among challenges in the development of TRPA1 antagonists is a notable species difference due to which some TRPA1 antagonists may be inactive in rodent TRPA1 that effectively prevents preclinical efficacy and safety studies [123]. On the other hand, the finding that the percentage of TRPA1 expressing human dorsal root ganglion neurons (80%) is much higher than that of mouse dorsal root ganglion neurons (40%) [124] raises the possibility that preclinical rodent studies may underestimate the potential efficacy of TRPA1 antagonists in clinical pain treatment. Various other challenges in the drug discovery process of TRPA1 antagonists have been briefly reviewed elsewhere [125].

Several pharmaceutical companies have actively been working on novel small molecule TRPA1 antagonists and optimizing existing scaffolds. Hydra Biosciences was the first company to disclose xanthine derivatives as TRPA1 antagonists, of which HC-030031 is a representative compound [126]. HC-030031 proved to be efficacious in vivo at 100 and 300 mg/kg. Another xanthine derivative Chembridge-5861528 was explored by Orion Pharma as a tool compound. It was shown to be about 10-fold more potent than HC-030031 [58]. Abbott disclosed a highly potent oxime derivative A-967079 (WO/2009/089082) that failed to show efficacy after oral administration in neuropathic pain models [127]. This result, however, may be explained by a limited access of A-967079 to the brain/spinal cord [9]. Amgen has disclosed a series of trichloro(sulfanyl)ethyl benzamides as potent TRPA1 antagonists, that were shown to be active only in human but not in rat TRPA1 [123]. Glenmark Pharmaceuticals has extensively characterized and expanded the chemical space identified by Hydra Biosciences. The most advanced compound developed by Glenmark Pharmaceuticals is GRC17536. For other compounds and their structures developed by various companies, see a recent review elsewhere [125].

Concerning testing of TRPA1 antagonists in humans, Hydra Biosciences/Cubist Pharmaceuticals have reported about a successful phase 1 study with their compound CB-625. However, the development of CB-625 was discontinued by Cubist Pharmaceuticals due to its low solubility. Orion Pharma conducted a phase 1 study with ODM-108, a highly potent TRPA1 antagonist with preference for human over rat TRPA1 receptors. This study was terminated due to complex pharmacokinetics. There were no safety concerns at the doses administered (adverse effects, vital signs, measures of sedation; NCT02432664 @ clinicaltrials.gov). Glenmark Pharmaceuticals has performed a phase 2 study in patients suffering from PDN and asthma. Patients with PDN may be classified into several subgroups based on their somatosensory phenotype [128]. In a Glenmark study, a peripherally restricted TRPA1 antagonist proved to be effective particularly in “an irritable nociceptor subgroup” of diabetic patients, in whom the symptoms are likely to be peripherally driven (WO/2016/042501). This result leaves open the question whether a TRPA1 antagonist that spreads to the central nervous system might be effective in a subgroup of patients in whom central mechanisms contribute to the symptoms, such as presumably in those with tactile allodynia. It is to be expected that with the development of novel TRPA1 antagonists the properties of which are optimal for clinical use, there will be more human studies allowing more conclusive interpretations on their role in clinical pain treatment. 

## 15. Conclusions

TRPA1 is a final common pathway for many pronociceptive agonists generated in various pathophysiological pain conditions, and for this reason it is a promising pain treatment target. In experimental animal studies, blocking TRPA1 has effectively attenuated pain behavior in many pathophysiological pain conditions. Depending on whether the TRPA1-mediated pronociception is due to enhanced transduction in the periphery, amplification of transmission centrally or both, pain treatment with a TRPA1 antagonist requires a compound that acts peripherally and/or centrally. Concerning PDN, treatment with a centrally acting TRPA1 antagonist alone is expected to treat patients with irritable nociceptor phenotype as well as a patient subgroup that suffers from mechanical hyperalgesia; i.e., 75% of PDN patients [129]. Prolonged blocking of TRPA1 has even had a disease-modifying effect in PDN, as shown by a delayed loss of a nociceptive nerve endings and their function in experimental diabetes. While the first phase 1 and 2 studies in humans have given some promising results e.g., in a subpopulation of PDN patients, additional study populations and probably also novel TRPA1 antagonists still need to be tested before making conclusive interpretation about the potential role of TRPA1 antagonists in clinical pain treatment.
